# A retrospective multi‐center feasibility study of a new PTV margin estimation approach for moving targets using CyberKnife log files

**DOI:** 10.1002/acm2.13975

**Published:** 2023-04-01

**Authors:** Payam Samadi Miandoab, Shahyar Saramad, Saeed Setayeshi, Oliver Blanck

**Affiliations:** ^1^ Department of Energy Engineering and Physics Medical Radiation Engineering Group, Amirkabir University of Technology Tehran Iran; ^2^ Department of Radiation Oncology University Medical Center Schleswig‐Holstein Kiel Germany

**Keywords:** CyberKnife system, errors, Pearson correlation, PTV margins, uncertainty

## Abstract

**Purpose:**

This study investigates a new approach for estimating the planning target volume (PTV) margin for moving tumors treated with robotic stereotactic body radiotherapy (SBRT).

**Methods:**

In this new approach, the covariance of modeling and prediction errors was estimated using error propagation and implemented in the Van Herk formula to form a Modified Van Herk formula (MVHF). To perform a retrospective multi‐center analysis, the MVHF was studied using 163 patients treated with different system versions of robotic SBRT (G3 version 6.2.3, VSI version 8.5, and VSI version 9.5) and compared with two established PTV margins estimation methods: The original Van Herk Formula (VHF) and the Uncertainty Estimation Method (UEM).

**Results:**

Overall, the PTV margins provided by the three formalisms are similar with 4–5 mm in the lung region and 4 mm in abdomen region to the PTV margins used in clinical. Furthermore, when analyzing individual patients, a difference of up to 1 mm was found between the PTV margin estimations using MVHF and VHF. The corresponding average discrepancies for the superior‐inferior (SI) direction ranged between −0.19 mm to 0.38 mm in CK G3 version 6.2.3, −0.36 mm to 0.33 mm in CK VSI version 8.5, and −0.34 mm to 0.40 mm in CK VSI version 9.5.

**Conclusions:**

It was found that for the lower left lung, upper left lung, lower right lung, upper right lung, central liver, and upper liver, the effect of covariance between model and prediction errors in SI direction was around 20%, 30%, 25%, 25%, 25%, and 30%, respectively. Notable covariance effects between model and prediction errors can be considered in PTV margin estimation using a modified VHF, which allowed for more precise target localization in robotic SBRT for moving tumors. Overall, in each of the three directions, the difference between MVHF and utilized clinical margins is 0.65 mm in the lung and abdominal region. Therefore, to improve the clinical PTV margins with the new approach, it is suggested to use the adaptive PTV margins in the next fractions.

## 1 INTRODUCTION

Stereotactic body radiation therapy (SBRT) is a cornerstone of cancer treatment, delivering high radiation doses precisely to tumor volumes while limiting exposure to surrounding healthy tissue.^1^, ^2^ One of the main challenges for SBRT is tumor motion caused by the patient's breathing. Currently, various special techniques, such as the breath‐holding technique, internal target volume (ITV), respiratory gating, and real‐time tumor‐tracking have been proposed for respiratory motion management.^3^, ^4^ In this series, the Synchrony Respiratory Tracking System (SRTS) integrated within the CyberKnife (CK) system (SRTS, Accuray Inc., Sunnyvale, CA, USA) is one option for performing SBRT of moving tumors. The CK system, an image‐guided system with a compact 6 MV linear accelerator (Linac) mounted on a robotic manipulator, is a dedicated SBRT system that can be used to treat a variety of tumors throughout the body with the required sub‐millimeter accuracy.^5^, ^6^ During treatment, the Linac can be precisely positioned using the 6 degrees (6D) of freedom robotic arms (3 for translations and 3 for rotations), while patients can breathe normally and the SRTS manages the target motion. The SRTS of the CK system includes a hybrid model that combines continuous external respiratory motion monitoring with sparse internal target position information to estimate tumor location during treatment. The external respiratory motion is detected by the three optical markers (light‐emitting diode (LED)) attached to the patient's chest, while the tumor location is detected by the x‐ray imaging system. Important advances in imaging and robotics of CK lead to target tracking using either the Fiducial‐based Target Tracking (FTT) or the Xsight Lung Tracking (XLT) system,^7^, ^8^ which both target tracking methods can be coupled with SRTS. The FTT system requires fiducial markers implanted near or inside the tumor, while the XLT system allows for direct tumor tracking without the need for implanted fiducials.^7^, ^8^ Of note, the XLT algorithm does not correct rotations. In addition, a prediction model in the SRTS is used to overcome system latency, which requires the estimation of the target location 115 ms (newer robot version) or 200 ms (robot version G3) into the future throughout treatment.^5^, ^6^

In general, error assessment is necessary for the CK to study the impact of delivery uncertainties and select appropriate PTV margins. In this context, a review of previous studies reveals several sources of error.^7^, ^8^, ^9^, ^10^, ^11^, ^12^, ^13^, ^14^, ^15^, ^16^, ^17^ Nakayama et al. and Descovich et al. found that the PTV margin estimation requires the consideration of STRS modeling and prediction errors.^7^, ^9^ According to Yang et al. study, the CK system has five main sources of error.^14^ 1) The segmentation error is the geometric difference in the relative positions of the tracked target (the tumor itself or implanted fiducials) in the digitally reconstructed radiographs (DRRs) and live x‐ray images. Note that, when the SRTS is enabled, the segmentation error is evaluated for the respiratory phase in which planning CT images were acquired (usually expiration). 2) In XLT, the segmentation error stands for the discrepancy between the relative positions of arbitrary points generated in the target volume and the target's center of mass (CoM) in real‐time x‐ray and planning CT images. Of note, the deformation error is estimated in the FTT by the difference in the location of the fiducials CoM in DRRs and live kV images, where a so‐called rigid body error is defined instead. 3) The modeling error is the difference between the correlation model's output and the tumor location as indicated by the x‐ray images. 4) The prediction error is the difference between the prediction algorithm and the associated correlation model 115 ms in the future. 5) The targeting error is the difference between the intended target position and where CK's robotic arm actually delivered the external beam.^14^ In this regard, details can be found in Floriano et al.,^12^ Chan et al.,^11^ Nakayama et al.,^7^ Pantelis et al.,^15^ Yang et al.,^14^ and Samadi et al.^16^

To explore the influence of uncertainties on estimated PTV margins, the cited studies employed the Uncertainty Estimation Method (UEM) or the Van Herk Formulation (VHF).^7^, ^9^, ^12^, ^14^ However, the previous studies highlighted that all error and PTV margin estimations are limited by small sample sizes. Furthermore, no consensus or study has been published to investigate the impact of the modeling and prediction errors covariance on the estimated PTV margins.

In this study, we are now considering the relationship between the standard deviation (SD) of the modeling and prediction errors, which allows for the inclusion of the covariance into a Modified Van Herk formulation (MVHF). We then evaluated the PTV margins for a large patient cohort (555 treatment fractions with FTT and XLT tracking in various locations). Finally, a comprehensive comparative study was conducted using the treatment log files from several versions of the CK (G3 version 6.2.3, VSI version 8.5, and VSI version 9.5) to compare the proposed formalism (MVHF) against two established PTV margins recipes (VHF and UEM). In this regard, a statistical analysis test was performed to verify the statistical significance of the estimated margins based on MVHF, VHF, and UEM.

## 2 MATERIALS AND METHODS

### 2.1 Data source and properties

This study evaluated the treatment log files of 163 patients with 555 treatment fractions. These datasets include 40 cancer patients (129 treatment fractions) treated with the CK G3 system version 6.2.3 at Georgetown University Hospital (Washington D.C., USA), 94 cancer patients (329 treatment fractions) treated with the CK VSI system version 8.5 at the University Medical Center Schleswig Holstein (Kiel, Germany), and 29 cancer patients (97 treatment fractions) treated with the CK VSI system version 9.5 at University Hospital Frankfurt (Germany). Treatment characteristics, including tumor locations, number of patients, treatment fractions, and target tracking system, are shown in Table 1. Of note, there are no technical differences regarding SRTS between CK Version 8.5 and CK version 9.5. In this study, to highlight the differences in the SRTS in the CK system, we split the results between various CK versions to show the possible clinically relevant differences.

**TABLE 1:** Case study characteristics, including tumor sites, number of patients, number of treatment fractions, and target tracking system for different versions of the CyberKnife system.

		CyberKnife G3 system with delivery software version 6.2.3			CyberKnife VSI system with delivery software version 8.5			CyberKnife VSI system with delivery software version 9.5		
Area	Tumor sites	Number of patients	Number of treatments	FTT or XLT system	Number of patients	Number of treatments	FTT or XLT system	Number of patients	Number of treatments	FTT or XLT system
Lung area	Lung apex left	1	3	FTT system	–	–	–	–	–	–
	Lower left lung	2	10	FTT system	10	29	XLT system	2	7	XLT system
	Upper left lung	11	33	FTT system	10	30	XLT system	3	10	XLT system
	Lower right lung	5	15	FTT system	10	29	XLT system	2	6	XLT system
	Central right lung	4	13	FTT system	10	37	XLT system	–	–	–
	Upper right lung	4	15	FTT system	10	32	XLT system	7	23	XLT system
	Central left lung	–	–	–	10	31	XLT system	–	–	–
Abdomen area	Liver	2	6	FTT system	–	–	–	–	–	–
	Central liver	–	–	–	10	35	FTT system	6	22	FTT system
	Lower liver	–	–	–	7	27	FTT system	–	–	
	Upper liver	–	–	–	10	45	FTT system	9	29	FTT system
	Pancreas	9	28	FTT system	7	34	FTT system	–	–	–
Chest wall	Chest wall	2	6	FTT system	–	–	–	–	–	–
Sum		40	129	–	94	329	–	29	97	–

Fiducial‐based Target Tracking (FTT) and 2‐view Xsight Lung Tracking (XLT) systems.

### 2.2 PTV margins estimation based on VHF, MVHF, and UEM

Previous research has demonstrated how the Van Herk formulation^18^, ^19^ (Equation 1) can be applied to derive a margin recipe for SBRT of lung cancer patients (Equation 2).^9^

(1) Margin=2.5∑+0.7σ

(2) $$\mathrm{Margin}\;=\;2.5\;\sum +\;\beta \sqrt{\sigma^{2}+\sigma_{\rho }^{2}}-\beta *\sigma_{\rho }$$

with the prescribed margins to ensure that the clinical target volume (CTV) receives at least 95% dose coverage for 90% of the patients when all errors and uncertainties are included, ∑ is the SD of individual systematic errors (the SD of the means and is an assessment of the treatment preparation's reproducibility), σ is the individual random error (the root mean square of the SD of the daily measurements), the penumbra width of the beam is represented by $\sigma_{\rho }$, which is determined by the dose level (β). The β is also the distance between the 95% and 50% isodose surface of the blurred dose distribution due to target motion. The radiation beam's penumbra width in the SBRT of the lung is wider than water, and because of the high dose fall‐off, an 80% dose level can be used in the dose prescription.^18^ For all calculations, including tumor located either in the lung or abdomen, $\sigma_{\rho }$ and β are assumed to be 6.4 mm and 0.84, respectively, under these conditions.

In addition to that, the results of the Yang et al.^14^ and Samadi et al.^16^ studies show how the non‐linear recipe (Equation 2) can be extended based on the five errors in the CK (Equation 3).^14^, ^16^

(3) $$\begin{array}{ccc} & & \mathrm{Margin}\;=\;2.5\\ & & \;\;\sqrt{{\left(\sum Seg\right)}^{2}+{\left(\sum Def\right)}^{2}+{\left(\sum Mod\right)}^{2}+{\left(\sum Pred\right)}^{2}+{\left(\sum Tar\right)}^{2}}\\ & & \;\;+\beta \sqrt{\sigma_{Mod+Pred}^{2}+\sigma_{\rho }^{2}}-\beta *\sigma_{\rho } \end{array}$$

(4) $$\;\sigma_{Mod+Pred}^{2}=\sigma_{Mod}^{2}\;+\;\sigma_{Pred}^{2}$$

where ∑Seg is the SD of the segmentation error, ∑Def is the SD of the deformation error, ∑Mod is the SD of the model error, ∑Pred is the SD of the prediction error, and ∑Tar is the SD of the targeting error from the robot precision. Noted that in deriving Equation 4, the SD of the combination of model error ($\sigma_{Mod}$) and prediction error ($\sigma_{Pred}$) is considered to be independent. However, based on error propagation,^20^, ^21^ the effect of random errors ($\sigma_{Mod}$ and $\sigma_{Pred}$) and their combination can be calculated and added to the main equation using the covariance value ($Cov\;(\sigma_{Mod},\sigma_{Pred})$). Therefore, a new recipe for the SD of the combined errors (model and prediction errors) can be proposed, which is:

(5) $$\begin{array}{c} \;\sigma \_new_{Mod+Pred}^{2}=\;\sigma_{Mod}^{2}\;+\;\sigma_{Pred}^{2}+2*Cov\;\left(\sigma_{Mod},\sigma_{Pred}\right) \end{array}$$

Also, the covariance value ($Cov\;(\sigma_{Mod},\sigma_{Pred})$), which refers to the covariance value between the SD of the model and prediction errors, is defined as follows:

(6) $$Cov\;\;\left(\sigma_{Mod},\sigma_{Pred}\right)=\;Corr\left(\sigma_{Mod},\sigma_{Pred}\right)*\sigma_{Mod}*\sigma_{Pred}$$

where the $Corr(\sigma_{Mod},\sigma_{Pred})$ is the correlation coefficient between the SD of the combination of model and prediction errors. Overall, in relation to covariance measure, three important concepts must be mentioned: positive covariance, negative covariance, or zero covariance indicating that two variables (model and prediction errors) move in the same direction, move in the opposite direction, or independently, respectively. Based on this fact, in this study, the Modified Van Herk formulation (MVHF) was proposed (Equation 7), in which the covariance value ($Cov(\sigma_{Mod},\sigma_{Pred})$) was the key quantity in this estimation.

(7) $$\begin{array}{ccc} & & M\;=\;2.5\\ & & \;\sqrt{{\left(\sum Seg\right)}^{2}+{\left(\sum Def\right)}^{2}+{\left(\sum Mod\right)}^{2}+{\left(\sum Pred\right)}^{2}+{\left(\sum Tar\right)}^{2}}\\ & & \;+\beta \sqrt{\sigma_{Mod}^{2}+\sigma_{Pred}^{2}+2.Cov\left(\sigma_{Mod},\sigma_{Pred}\right)+\sigma_{\rho }^{2}}-\beta *\sigma_{\rho } \end{array}$$

The model error based on the x‐ray images taken during treatment is available approximately every minute, and the prediction error is available every 40 ms during treatment delivery. Therefore, the covariance value can be estimated from the data sets when updating the model. In order to estimate the effect of covariance between different error types for the total number of treatment fractions, the maximum correlation and covariance values were first estimated for all fractions of a patient. Then, the average of the maximum correlation, and covariance values was estimated for the total number of patients. The final results are shown in Table S1. According to this table, the average correlation values for different patients are in the range of −0.23 to 0.31. Since the SD of the model and prediction errors can also affect the covariance value, the direction of maximum average covariance may not coincide with the direction of maximum average correlation. It must be noted that the relationship between model and prediction errors (covariance value) in the first fraction is critical, as it could be used for adaptive PTV margin calculation in subsequent fractions.

Floriano et al.^12^ proposed a new approach (the UEM technique) that involves four main steps to determine the position of 95% of CTV points with a 95% confidence level during treatment based on the source of uncertainties, including segmentation, deformation, correlation, prediction, and robotics. These steps will not be repeated in this study.

The segmentation error is defined as the difference between the tracked target's apparent positions in the live x‐ray images and DRRs, and it is subject to differences in imaging parameters (kVp, mAs). Distinguishing the segmentation error in the real‐time x‐ray or DRR images is difficult due to the poor soft‐tissue contrast of CT images and tumor density limitations. In this regard, Jung et al.^22^ used patient‐specific lung phantoms and reported an SD of segmentation error (∑Seg) of 0.54 mm in all three directions. In this study, the segmentation error is assigned based on Jung et al. study.^22^ On the other hand, the deformation error depends on individual patient features (tumor size, location, and motion range). In this relation, Smith et al.^23^ and Lu et al.^24^ conducted a study to calculate the deformation error in lung tumors. Smith et al.^23^ investigated the deformation error and plotted it as a function of tumor centroid distance (figure 5 in Smith et al.^23^). They also report that the deformation error increases with increasing distance from the CoM, and that 3 mm margins are required to cover this effect. Also, Lu et al.^24^ found that a 3 mm margin can cover the deformation error while keeping CTV dose coverage above 95%.

In this study, based on tumor size, location, and motion range, the deformation error is defined as follows: 1) if the tumor motion, determined by its CoM, is less than 2 cm, the uncertainty of the deformation error is considered to be 1.5 mm in all three directions; 2) 2.5 mm if the tumor motion is more than 2 cm. Also, the CK's targeting error was measured using E2E tests. Note that, for CK 8.5 and 9.5, the E2E test results were 0.37 ± 0.53 mm in the superior‐inferior (SI) direction, 0.12 ± 0.19 mm in the left‐right (LR) direction, and 0.15 ± 0.36 mm in the anterior‐posterior (AP) direction, while the reported E2E for CK G3 was 0.5 ± 0.3 mm in all three directions.^16^

### 2.3 Implemented scenarios for validation MVHF

To better understand the impact of covariance values on MVHF, two scenarios were considered. First, the treatment of individual patients with CK 8.5 in different tumor locations and directions was examined. In this case, the estimated PTV margin based on the VHF formula in the first fraction was compared with the MVHF in the next fractions. It should be noted that the covariance value was extracted from the data sets available in the first fraction during treatment to estimate the PTV margins in the following fractions using the MVHF method. Overall, the aim of the first scenario is to find out the advantage of using the information from the first fraction to adjust the PTV margins in the next treatment fractions. Second, the maximum and minimum covariance values were estimated for each patient in each tumor location and direction. In this regard, Equation (8) estimates the relative difference (%) between the Van Herk and Modified Van Herk PTV margins for the maximum and minimum correlation values.

(8) $$\begin{array}{ccc} & & \mathrm{Relative}\;\mathrm{Difference}\;\;\left(\%\right)\\ & & =\frac{\left(\mathrm{Modified}\;\mathrm{Van}\;\mathrm{Herk}\;\mathrm{PTV}\;\mathrm{Margin}-\mathrm{Van}\;\mathrm{Herk}\;\mathrm{PTV}\;\mathrm{Margin}\right)}{\mathrm{Van}\;\mathrm{Herk}\;\mathrm{PTV}\;\mathrm{Margin}}\\ & & *100 \end{array}$$

### 2.4 Statistical analysis

In this study, statistical F‐test analysis was performed for all tumor locations and directions to test for a statistically significant relationship between estimated margins based on VHF, MVHF, and UEM. A *p*‐value of 0.05 or less was considered statistically significant. Despite the fact that Pearson's correlation $Corr(\sigma_{Mod},\sigma_{Pred})$ in Equation 6 evaluates a linear relationship between model and prediction errors, there is no guarantee that this relationship is linear. Consequently, a non‐linear correlation analysis must also be investigated. In this context, distance correlation is an excellent measure of a non‐linear relationship between two variables, which is defined as follows:

(9) $$dCorr\;\left(\sigma_{Mod},\sigma_{Pred}\right)=\;\frac{dCov^{2}\;\left(\sigma_{Mod},\sigma_{Pred}\right)}{\sqrt{dVar_{\left(Mod\right)}*dVar_{\left(Pred\right)}}}\;$$

where $dCorr(\sigma_{Mod},\sigma_{Pred})$ is the distance correlation between model and prediction errors, dCov(Mod,Pred) is distance covariance between model and prediction errors, $dVar_{\left(Mod\right)}$ is the distance variance of the model error, and $dVar_{\left(Pred\right)}$ is distance variance of the prediction error. Overall, the distance correlation ranged from 0 to 1, where 0 implies independence between two variables.^25^

## 3 RESULTS

**FIGURE 1:**
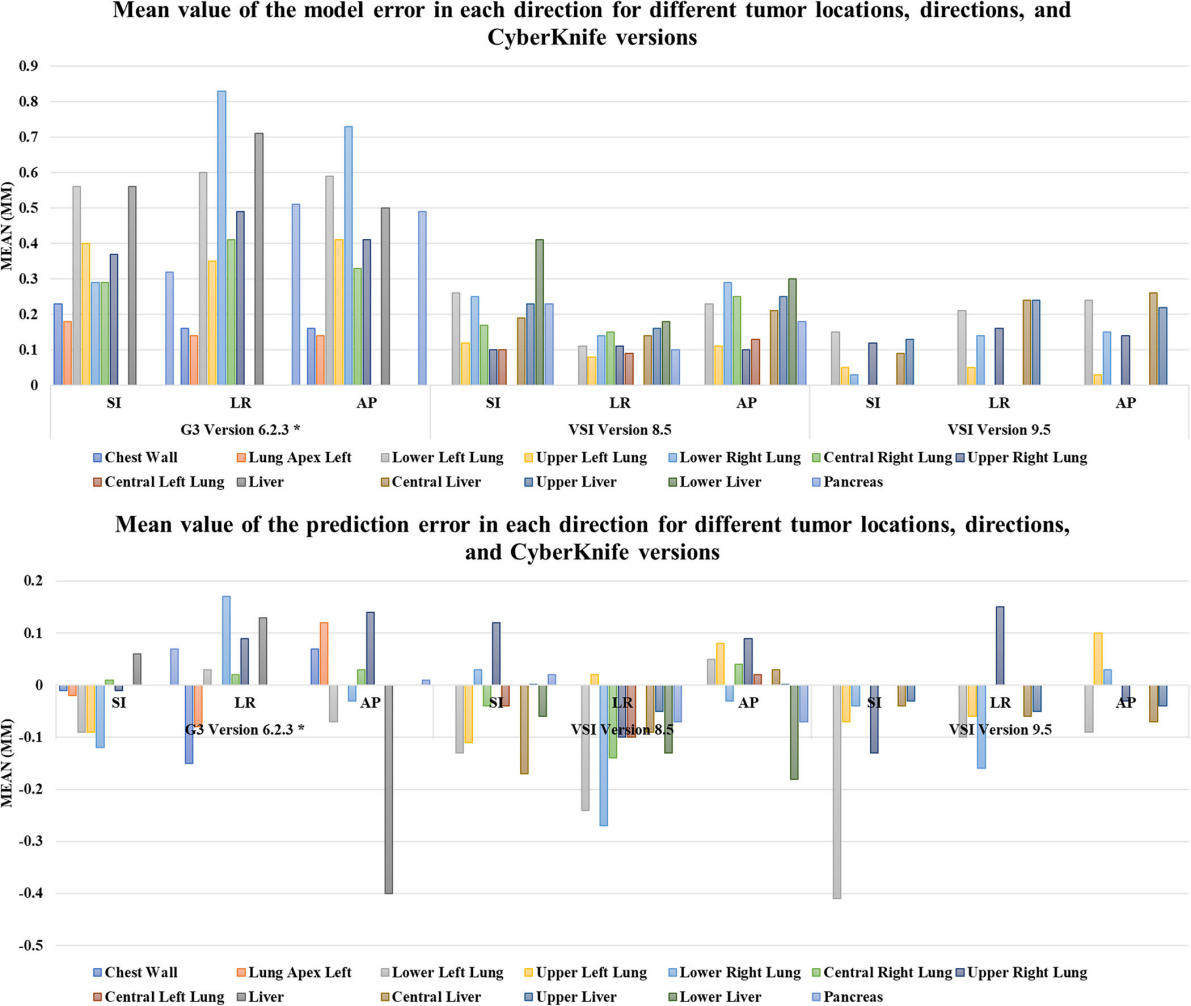
The mean of the model (top) and prediction (bottom) errors in each direction for different tumor locations and CyberKnife versions.

**FIGURE 2:**
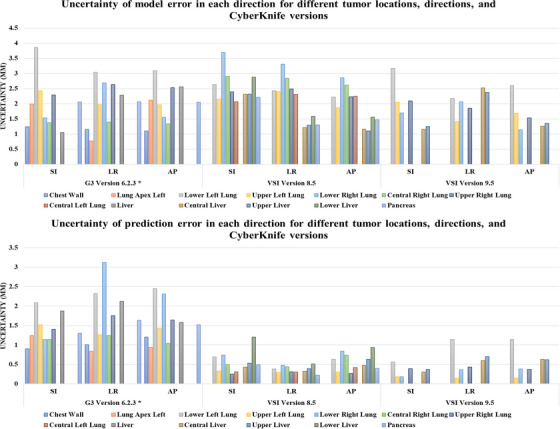
Uncertainty of model (top) and prediction (bottom) errors in each direction for different tumor locations and CyberKnife versions.

The results of errors and uncertainties for different tumor locations, directions, and CK versions are summarized in Figures 1 and 2. Detailed information about the reported error and uncertainty values can also be found in Tables S2 and S3, respectively. An analysis of the tracking information of the SRTS in different versions of the CK shows that the modeling uncertainty is the dominant source of error. On the other hand, due to an improvement in the prediction algorithms, the prediction error is significantly reduced compared to the CK G3 system. It is important to note that uncertainties in fitting model types (a linear, quadratic, or constrained fourth‐order polynomial estimation model) due to various types of internal/external motion patterns, such as linear motion, non‐linear motion, or hysteresis, and internal‐external datasets, which are respectively related to external optical tracking and x‐ray imaging system, cause the model error to be dominant throughout treatment. Furthermore, regions near the diaphragm with larger displacements have significantly higher uncertainties.

Overall, the mean and SD of the total tracking errors for the FTT (G3 version 6.2.3), which is the square root of the sum of the squares of the model, prediction, and E2E tracking errors, were 0.57 ± 1.42 mm in the SI direction, 0.59 ± 1.44 mm in the LR direction, and 0.56 ± 1.33 mm in the AP direction for lung targets. The values for the XLT (VSI version 8.5) were 0.44 ± 1.33 mm, 0.24 ± 1.20 mm, and 0.25 ± 1.10 mm, and for the XLT (VSI version 9.5) were 0.44 ± 1.16 mm, 0.23 ± 0.90 mm, and 0.22 ± 0.90 mm in the SI, LR, AP directions, respectively. Comparing the target tracking system in a large sample shows no significant difference between the two tracking systems (FTT and XLT systems). Note that the CK version, system latency, patient selection, tumor location, estimation model types, and prediction software version contributed to the target tracking performance differences.

By using Equations 6 and 9, the linear correlation (Pearson correlation) and non‐linear correlation (Distance correlation) values between two variables (model and prediction errors) were tested, respectively. In this regard, four lung regions, including the lower left lung, upper left lung, lower right lung, and upper right lung, were examined separately to investigate this relationship. Table S4 results show that for the CK G3 version 6.2.3, the average Pearson and distance correlations were in the range of −0.14 to 0.20 and 0.17 to 0.38, respectively. Those values were −0.24 to 0.21 and 0.19 to 0.41 for the CK VSI version 8.5, were −0.21 to 0.24 and 0.14 to 0.39 for the CK VSI version 9.5. Since the distance correlation is always larger than the Pearson correlation, a non‐linear behavior is expected in all versions of the CK system (Table S4). But it must be noted that in the present work, the estimated modified VH formula is based on Pearson correlation and not distance correlation. So, for studying the effect of distance correlation (non‐linear behavior) on the estimated PTV margin, the MVHF must be also extended, which is beyond the scope of this work.

The effect of the covariance values on the MVHF and its comparison with the established PTV margins estimation methods (VHF and UEM) is shown in Table 2. Since the covariance values for different patients in a region can have positive or negative values, their net effect can be significantly reduced by averaging, which can be seen in Table 2. Therefore, the average covariance values in different regions and directions have a small effect on the MVHF. The presented strategy for extracting the covariance and linear correlation values for each patient assumes that the correlation values remain constant through the total number of treated fractions. Furthermore, according to statistical analysis, the calculated *p*‐values in the liver region of the CK G3, considering the estimated PTV margins for the three methods (VHF, MVHF, and UEM) were (*p* = 0.039, *p* = 0.036, and *p* = 0.041) in the SI direction, (*p* = 0.028, *p* = 0.027, and *p* = 0.028) in the LR direction, and (*p* = 0.023, *p* = 0.021, and *p* = 0.025) in the AP direction, respectively. In the CK VSI‐version 9.5, for the VHF, MVHF, and UEM methods, the *p*‐values for the estimated PTV margins were (*p* = 0.041, *p* = 0.037, and *p* = 0.040) in the SI direction, (*p* = 0.030, *p* = 0.027, and *p* = 0.030) in the LR direction, and (*p* = 0.025, *p* = 0.021, and *p* = 0.024) in the AP direction, respectively, and only a slight discrepancy in the central liver region observed.

**TABLE 2:** The mean ± SD of the calculated PTV margins based on VHF, MVHF, and UEM in each direction for different tumor locations and CyberKnife versions

			Tumor sites												
CyberKnife type			Chest wall	Lung apex left	Lower left lung	Upper left lung	Lower right lung	Central right lung	Upper right lung	Central left lung	Liver	Central liver	Upper liver	Lower liver	Pancreas
G3 version 6.2.3	PTV margin based on VHF [mm]**^a^**	SI	2.65 ± 0.40	4.14 ± 1.01	4.87 ± 1.70	4.30 ± 1.19	2.95 ± 0.70	2.97 ± 0.65	3.40 ± 1.52	–	2.88 ± 0.60	–	–	–	4.04 ± 0.71
		LR	2.73 ± 0.60	3.56 ± 0.33	4.61 ± 1.13	4.13 ± 1.21	3.67 ± 1.96	2.78 ± 0.69	3.97 ± 1.99	–	3.26 ± 0.51	–	–	–	4.10 ± 0.39
		AP	2.92 ± 0.98	4.05 ± 0.90	4.75 ± 1.55	4.13 ± 1.26	3.01 ± 0.91	2.70 ± 0.50	3.37 ± 0.97	–	3.22 ± 1.11	–	–	–	4.16 ± 1.05
	PTV margin based on MVHF [mm]	SI	2.70 ± 0.45	4.11 ± 1.08	5.06 ± 1.89	4.39 ± 1.25	3.14 ± 0.79	3.12 ± 0.69	3.50 ± 1.66	–	2.99 ± 0.65	–	–	–	4.07 ± 0.78
		LR	2.69 ± 0.52	3.55 ± 0.37	4.53 ± 1.04	4.17 ± 1.27	3.53 ± 1.83	2.75 ± 0.64	3.90 ± 2.1	–	3.36 ± 0.69	–	–	–	4.17 ± 0.45
		AP	2.91 ± 0.96	4.07 ± 0.94	4.64 ± 1.49	4.18 ± 1.29	3.09 ± 0.99	2.82 ± 0.56	3.30 ± 1.05	–	3.32 ± 1.19	–	–	–	4.14 ± 1.00
	PTV margin based on UEM [mm]**^a^**	SI	2.81 ± 0.46	4.01 ± 0.98	5.13 ± 1.35	4.29 ± 1.12	3.08 ± 0.55	2.97 ± 0.53	3.71 ± 1.51	–	3.07 ± 0.50	–	–	–	4.0 ± 0.69
		LR	2.91 ± 0.62	3.28 ± 0.26	5.02 ± 1.03	3.97 ± 1.01	4.81 ± 1.85	3.0 ± 0.51	4.15 ± 2.22	–	3.92 ± 0.41	–	–	–	4.08 ± 0.56
		AP	3.07 ± 1.07	3.93 ± 0.75	5.06 ± 1.62	4.03 ± 0.90	3.68 ± 0.76	2.87 ± 0.39	3.90 ± 1.04	–	3.88 ± 1.15	–	–	–	4.07 ± 0.89
VSI version 8.5	PTV margin based on VHF [mm]	SI	–	–	4.18 ± 0.44	4.09 ± 0.45	3.56 ± 0.57	3.35 ± 0.59	3.29 ± 0.88	4.09 ± 0.59	–	3.00 ± 0.36	2.95 ± 0.29	3.27 ± 0.40	2.89 ± 0.20
		LR	–	–	4.08 ± 0.37	4.07 ± 0.49	3.44 ± 0.71	3.22 ± 0.47	3.17 ± 0.26	4.04 ± 0.38	–	2.61 ± 0.18	2.65 ± 0.17	2.74 ± 0.18	2.69 ± 0.15
		AP	–	–	4.18 ± 0.70	3.83 ± 0.25	3.25 ± 0.43	3.38 ± 0.87	2.93 ± 0.23	4.13 ± 0.65	–	2.65 ± 0.15	2.60 ± 0.11	2.79 ± 0.23	2.65 ± 0.09
	PTV margin based on MVHF [mm]	SI	–	–	4.19 ± 0.46	4.02 ± 0.44	3.65 ± 0.52	3.30 ± 0.58	3.21 ± 0.84	4.05 ± 0.49	–	3.10 ± 0.35	2.99 ± 0.30	3.25 ± 0.37	2.99 ± 0.19
		LR	–	–	4.11 ± 0.38	4.12 ± 0.51	3.33 ± 0.70	3.25 ± 0.47	3.09 ± 0.27	4.04 ± 0.38	–	2.71 ± 0.18	2.75 ± 0.18	2.73 ± 0.18	2.62 ± 0.10
		AP	–	–	4.21 ± 0.75	3.89 ± 0.20	3.34 ± 0.46	3.41 ± 0.89	2.93 ± 0.23	4.23 ± 0.62	–	2.61 ± 0.15	2.60 ± 0.12	2.79 ± 0.22	2.67 ± 0.08
	PTV margin based on UEM [mm]	SI	–	–	4.12 ± 0.77	3.77 ± 0.79	4.45 ± 1.37	3.78 ± 1.24	3.36 ± 1.30	3.78 ± 1.06	–	3.29 ± 0.78	3.31 ± 0.75	3.90 ± 1.25	3.77 ± 0.44
		LR	–	–	3.96 ± 0.90	3.95 ± 1.01	4.14 ± 1.79	3.72 ± 1.11	3.38 ± 0.66	3.90 ± 0.82	–	2.58 ± 0.36	2.63 ± 0.39	2.82 ± 0.57	3.27 ± 0.19
		AP	–	–	3.93 ± 1.02	3.58 ± 0.47	3.87 ± 1.12	3.67 ± 1.44	3.22 ± 0.77	3.99 ± 1.62	–	2.59 ± 0.38	2.58 ± 0.27	2.98 ± 0.79	3.36 ± 0.18
VSI version 9.5	PTV margin based on VHF [mm]	SI	–	–	4.32 ± 0.31	3.99 ± 0.46	2.79 ± 0.30	–	2.93 ± 0.45	–	–	2.60 ± 0.18	2.63 ± 0.14	–	–
		LR	–	–	4.11 ± 0.46	3.76 ± 0.27	2.86 ± 0.23	–	2.77 ± 0.33	–	–	3.13 ± 0.29	2.98 ± 0.26	–	–
		AP	–	–	4.29 ± 0.64	3.85 ± 0.37	2.66 ± 0.16	–	2.78 ± 0.39	–	–	2.63 ± 0.10	2.65 ± 0.19	–	–
	PTV margin based on MVHF [mm]	SI	–	–	4.34 ± 0.31	3.98 ± 0.46	2.85 ± 0.30	–	2.99 ± 0.45	–	–	2.69 ± 0.11	2.63 ± 0.14	–	–
		LR	–	–	4.12 ± 0.48	3.71 ± 0.29	2.89 ± 0.24	–	2.87 ± 0.35	–	–	3.05 ± 0.21	2.99 ± 0.29	–	–
		AP	–	–	4.31 ± 0.67	3.95 ± 0.37	2.69 ± 0.19	–	2.83 ± 0.39	–	–	2.69 ± 0.10	2.69 ± 0.17	–	–
	PTV margin based on UEM [mm]	SI	–	–	4.47 ± 1.03	3.75 ± 0.83	2.88 ± 0.70	–	3.14 ± 0.91	–	–	2.54 ± 0.32	2.61 ± 0.38	–	–
		LR	–	–	4.12 ± 1.01	3.37 ± 0.50	3.09 ± 0.52	–	3.02 ± 0.81	–	–	3.44 ± 0.68	3.38 ± 0.68	–	–
		AP	–	–	4.42 ± 1.20	3.53 ± 0.62	2.61 ± 0.58	–	2.79 ± 0.74	–	–	2.66 ± 0.32	2.73 ± 0.46	–	–

All value is based on Mean value ± Standard Deviation.

Abbreviations: SI, Superior‐Inferior; LR, Left‐Right; AP, Anterior‐Posterior.

^a^ Reported by Samadi et al.^16^

**FIGURE 3:**
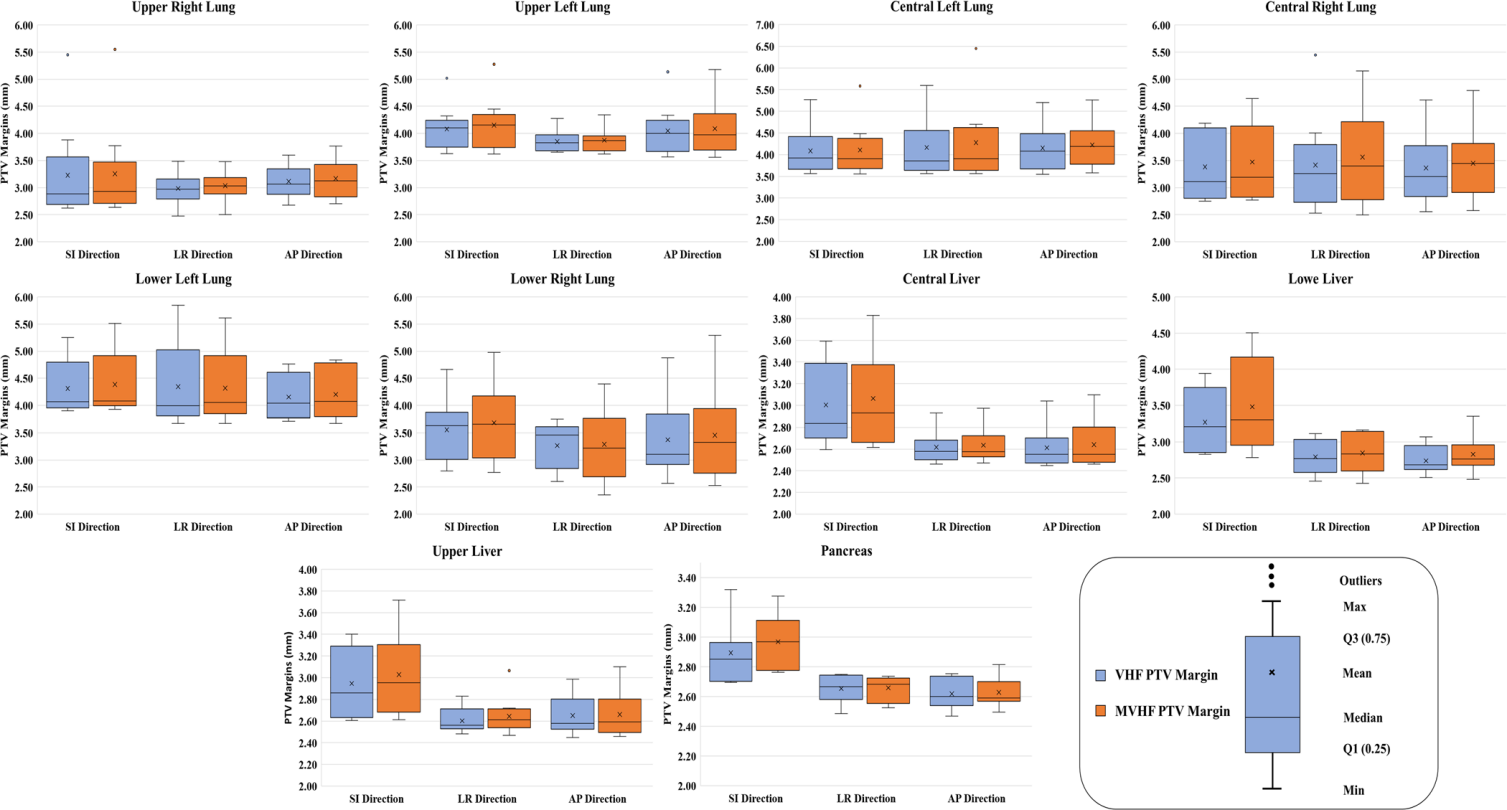
The boxplot of the comparison between the PTV margins estimated by the VHF in the first fraction and the MVHF in the next fraction for different tumor locations and directions treated with the CyberKnife VSI‐version 8.5. For each box, the horizontal line shows the median, the x mark shows the mean, and the bottom and top of the box show the 25th and 75th percentiles, respectively. Outliers are shown separately as a dot.

**FIGURE 4:**
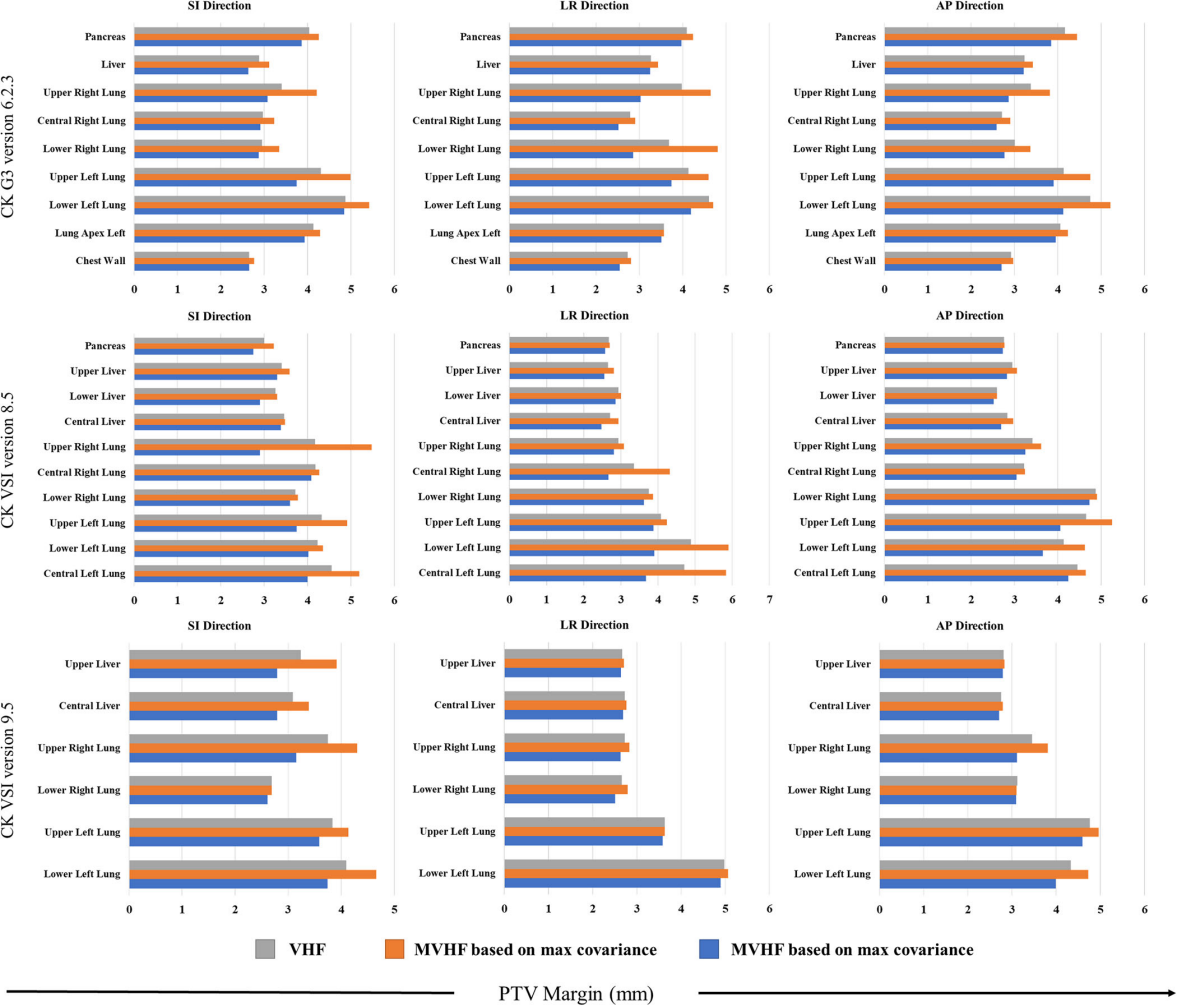
Estimated PTV margin based on VHF, MVHF based on max covariance, and MVHF based on min covariance in different tumor locations and directions of the CyberKnife G3 version 6.2.3, CyberKnife VSI version 8.5, and CyberKnife VSI version 9.5.

The results of the first scenario (the calculated PTV margins using VHF in the first fraction and the MVHF in the next fractions) and second scenario (the calculated PTV margin using VHF and MVHF based on max and min covariance) are also shown in Figures 3 and 4, respectively. The boxplot in Figure 3 compares the estimated PTV margins from the VHF in the first fraction with the MVHF in the second fractions for different tumor locations and directions treated with CK VSI version 8.5. On the other hand, Figure 4 compares the estimated PTV margin based on MVHF and VHF in the CK G3 version 6.2.3, CK VSI version 8.5, and CK VSI version 9.5. More information on the estimated maximum and minimum PTV margins for the modified VHF, as well as the relative difference (%), can be found in Table S5.1, S5.2, and S5.3 for G3 version 6.2.3, VSI version 8.5, and VSI version 9.5, respectively. This figure also allows a better comparison of the PTV margins reported by the VHF and MVHF by considering the range of covariance (the maximum and minimum covariance values) for each tumor location and direction. According to this figure, locations with larger displacements, such as the lower left lung, lower right lung region, and upper liver, have the largest relative differences. Therefore, for the estimation of the PTV margin in these regions, the covariance values must be considered.

## 4 DISCUSSION

A variety of SBRT techniques can be used to reduce the irradiated volume in the case of respiratory target motion.^26^ The Synchrony real‐time tumor tracking technology incorporated into the CK provides a high degree of precision in delivering the radiation dose.^27^ In this context, evaluating the SRTS is crucial to assess the precision of dose delivery. Several studies have attempted to identify the sources of uncertainties in the CK system and/or presented a formalism to estimate PTV margins.^7^, ^8^, ^9^, ^10^, ^11^, ^12^, ^13^, ^14^, ^16^ Descovich et al., for example, used the VHF in 24 lung cancer patients to determine the internal target volume (ITV) to PTV margins. Excluding the respiratory tracking system, the estimated ITV to PTV margins in this cohort was 6.81 mm, 4.42 mm, and 4.67 mm in the SI, LR, and AP directions, respectively.^9^ Floriano et al. developed an alternative technique based on the uncertainty level for calculating tumor margins. According to their results, 5 mm margins were sufficient in all three directions in 16 lung cancer patients treated with the FTT system.^12^ Chan et al. study residual tracking errors in liver metastasis (46 patients) and discover that a 3 mm margin may not always provide 95% coverage of all tracking errors, mainly due to uncompensated tumor rotation.^11^ Recently, Samadi et al. evaluated the clinical data (45 cancer patients) of the CK G3 system (FTT system) to determine PTV margins using two methods: VHF and UEM. They found that 4−5 and 5 mm margins in all three directions were sufficient to provide greater than 95% coverage for lung and abdominal tumors, respectively.^16^

In the present study, the analysis was conducted in two steps. The first step involved a comprehensive comparison of the performance of the FTT and XLT systems in different tumor locations, directions, and CK versions for 163 lung, liver, and pancreas cancer patients. Then, the effects of the correlation‐prediction‐error interaction levels (covariance value) were analyzed by considering a new term in the Van Herk formula for the PTV margin calculation for 555 treatment fractions. It should be noted that different from previous studies^10^, ^11^, ^16^ in this work liver tumors were divided into three groups (lower liver, central liver, and upper liver), and mean and SD of modeling and prediction errors as well as treatment uncertainties were estimated for each region. In addition, a comprehensive comparative analysis was performed between MVHF and two established PTV margin estimation methods (VHF and UEM).

From the results of this study, it can be seen that the three different formalisms resulted in similar PTV margins consistent with the results of other studies.^10^, ^12^, ^14^, ^16^ Additionally, the results of the three formalisms are consistent with the utilized clinical margins (5 and 3 mm, respectively) in the lung and abdominal areas. However, the covariance effect in MVHF can increase or decrease the estimated PTV margins depending on tumor locations and error directions (especially in the SI direction). Overall, using an average covariance value to calculate the margins shows a minimal effect on the calculated PTV margin (median line and average x‐marker in the boxplot in Figure 3) compared to the VHF; therefore, the first scenario of MVHF is proposed. In this regard, considering the SD of the boxplot in Figure 3 shows that the MVHF has a higher SD in the liver region, including the lower, middle, and upper livers, as well as the lower left and lower right lungs. Therefore, we propose the necessity for PTV margin estimations based on individual patient analysis. This is underlined by the result that average PTV margins (see Figure S1‐a and S1‐b) were not always sufficient to cover all tracking errors in all patients. For example, in 70% of patients in the lower left lung region, a 4 mm margin (on average) provides 95% coverage in all three directions, whereas, in 30% of patients, a margin of more than 5 mm is required. The same is true for other tumor locations. Furthermore, when considering the impact of the covariance value, the estimated PTV margins may be increased or decreased depending on the type of relationship (positive or negative correlation), which could be calculated from pre‐treatment simulation or after the first fraction. For example, the calculated PTV margin based on the VHF method was 5.6 mm in the LR direction in the first treatment fraction of patient 4 (central left lung), whereas, in the next treatment fractions, it was increased to 6.45 mm by considering the covariance effects. The same is true for the central right lung (patients 6, 7, and 8), lower left lung (patient 1), lower right lung (patients 5, 6, and 8), upper left lung (patient 2), upper right lung (patient 1), central liver (patient 2), lower liver (patients 4 and 6), upper liver (patients 3 and 7), and pancreas (patient 2). Overall, the proposed method (MVHF) differs 0.65 mm in three directions from the clinical margins used in the lung and abdominal regions.

Our findings show that the correlation values for different patients can be positive or negative (see Table S5‐1, S5‐2, and S5‐3), resulting in an average relative difference (%) in the PTV ranging from −17% to 23%, −12% to 7%, and −5% to 10% for all tumor locations and directions in the CK G3 version 6.2.3, CK VSI version 8.5, and CK VSI version 9.5, respectively. In this regard, Figure 4 shows that the corresponding average discrepancy in the CK G3 version 6.2.3 for all tumor locations ranged from −0.19 mm to 0.38 mm in SI, −0.36 mm to 0.32 mm in LR, and −0.27 mm to 0.31 mm in AP. This value ranged between −0.36 mm to 0.33 mm, −0.37 mm to 0.40 mm, and −0.21 mm to 0.18 mm for CK VSI version 8.5, and −0.34 mm to 0.40 mm, −0.07 mm to 0.07 mm, and −0.16 mm to 0.17 mm for CK VSI version 9.5 in SI, LR, and AP directions, respectively. Therefore, the largest relative difference (%) in the modified Van Herk PTV margin relates to the lower left lung, lower right lung, and central right lung in the lung regions (see Table S5‐1, S5‐2, and S5‐3).

Table 2 shows that for the CK G3 version 6.2.3 in all regions and directions, for 48% of the cases, the PTV margins extracted by the UEM are between the minimum and maximum PTV margins of the MVHF. For the other 33% of cases, the difference between the two PTV margins is less than 5%. In the VSI 8.5 and 9.5, the PTV margins extracted from the MVHF are between the minimum and maximum PTV margins of the UEM in 90% and 94.5% of the cases, respectively. Therefore, for the CK G3‐ version 6.2.3, the range of variation of the PTV margins of the UEM is smaller than that of the MVHF, but for the VSI version 9.5, it is larger than that of the MVHF. Overall, the PTV margins calculated using three proven techniques in the VSI version of CK (VSI versions 8.5 and 9.5) were clinically consistent with each other, and there was no statistically significant difference between these three different formalisms. However, by using the maximum correlation values, the MVHF has detailed information to more accurately extract the PTV margin for each patient, which is an advantage over the UEM.

The current study has three more limitations. 1) After the first treatment fraction, the MVHF method is valid. 2) The segmentation and deformation errors are extracted from previous studies. 3) The possibility of using a new method (MVHF) to estimate PTV margins was technically discussed. However, the practical implementation and clinical significance remain unknown, which was beyond the scope of this work and will be further investigated. It is also interesting to investigate the pseudo‐error dose calculation (dosimetric results) in order to better understand the relationship between dose and margin.

## 5 CONCLUSION

In this study, a novel technique was employed to construct a Modified Van Herk formula (MVHF) by first using error propagation to estimate the covariance of modeling and prediction errors. To the best of our knowledge, the new approach (MVHF) for estimating PTV margins was one of the first attempts to use the adaptive PTV margins in the next fractions. According to MVHF, the estimated PTV margins are more affected in the lower left lung, upper left lung, lower right lung, upper right lung, central liver, and upper liver around 20%, 30%, 25%, 25%, 25%, 25%, and 30% in SI direction, respectively. Overall, in the SI direction, the average of PTV margin deviations between MVHF and VHF for all tumor locations ranged from −0.19 mm to 0.38 mm, −0.36 mm to 0.33 mm, and −0.34 mm to 0.40 mm in CK G3 version 6.2.3, VSI 8.5, and VSI 9.5. In addition, there are 0.65 mm differences in three directions between the proposed approach (MVHF) and the clinical margins that were used in the lung and abdominal regions. Therefore, to utilize the new approach to improve the clinical PTV margins, it is suggested to use the adaptive PTV margins in the next fractions.

## AUTHOR CONTRIBUTIONS

All listed authors contributed to the study and to drafting the manuscript.

## CONFLICT OF INTEREST STATEMENT

The authors declare that they have no known competing financial interests or personal relationships that could have appeared to influence the work reported in this paper.

## ETHICAL APPROVAL STATEMENT

The Georgetown Institutional Review Board has approved the use of these data for research purposes (IRB‐2005‐309). The ethics committees of the University of Kiel and the University of Frankfurt have approved the use of anonymized data within the framework of the clinical evaluation of the Cyber‐Knife Synchrony System (KI D 494/17 and F 477/15).

## Supporting information

Supporting Information

Supporting Information

Supporting Information
