# Supplementary figures and images for: A retrospective multi‐center feasibility study of a new PTV margin estimation approach for moving targets using CyberKnife log files
Source: J Appl Clin Med Phys. 2023 Apr 1;24(7):e13975. doi: 10.1002/acm2.13975 (PMC10338771; doi:10.1002/acm2.13975)

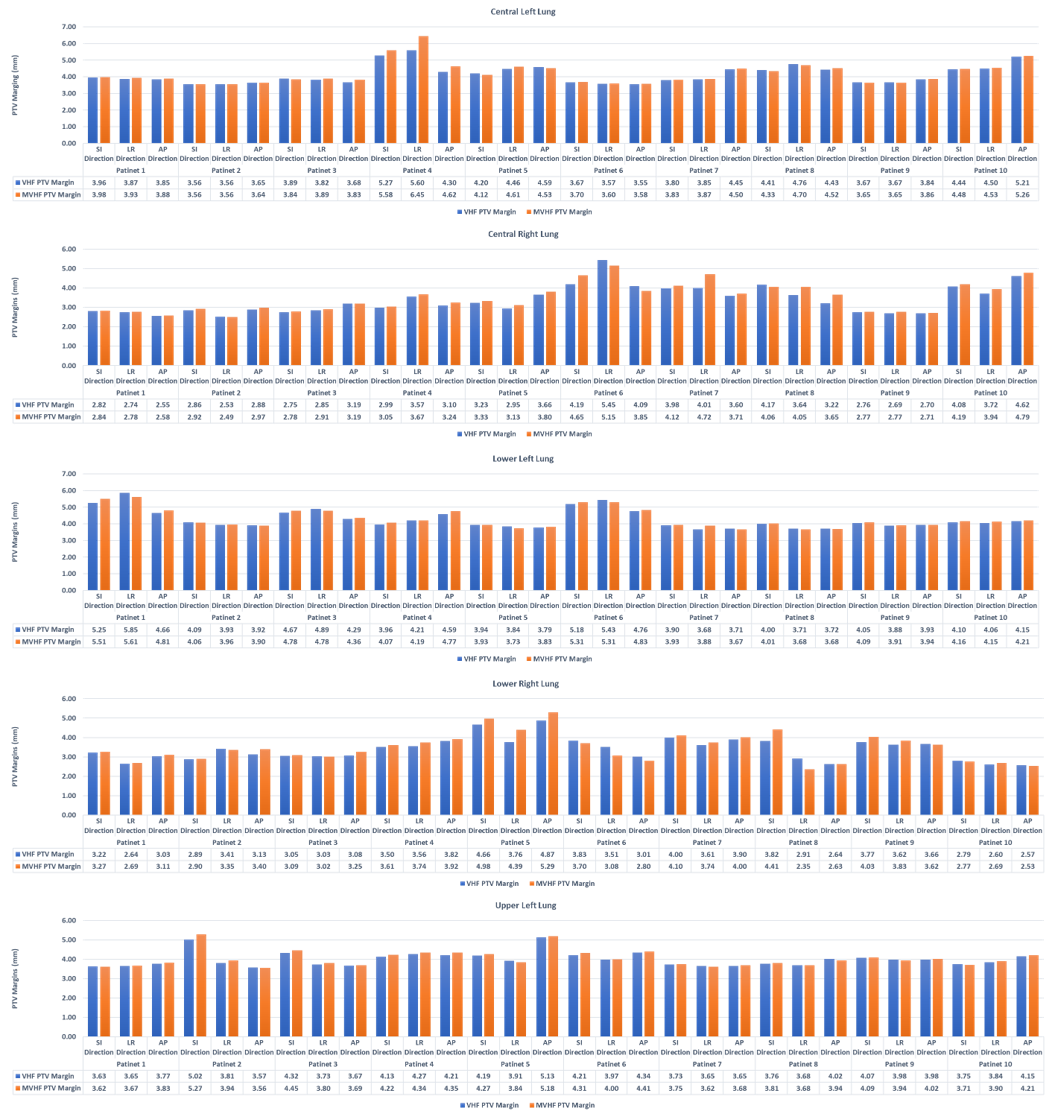

Supplement: Supplementary file 1 — Supporting Information [file ACM2-24-e13975-s001.tif]

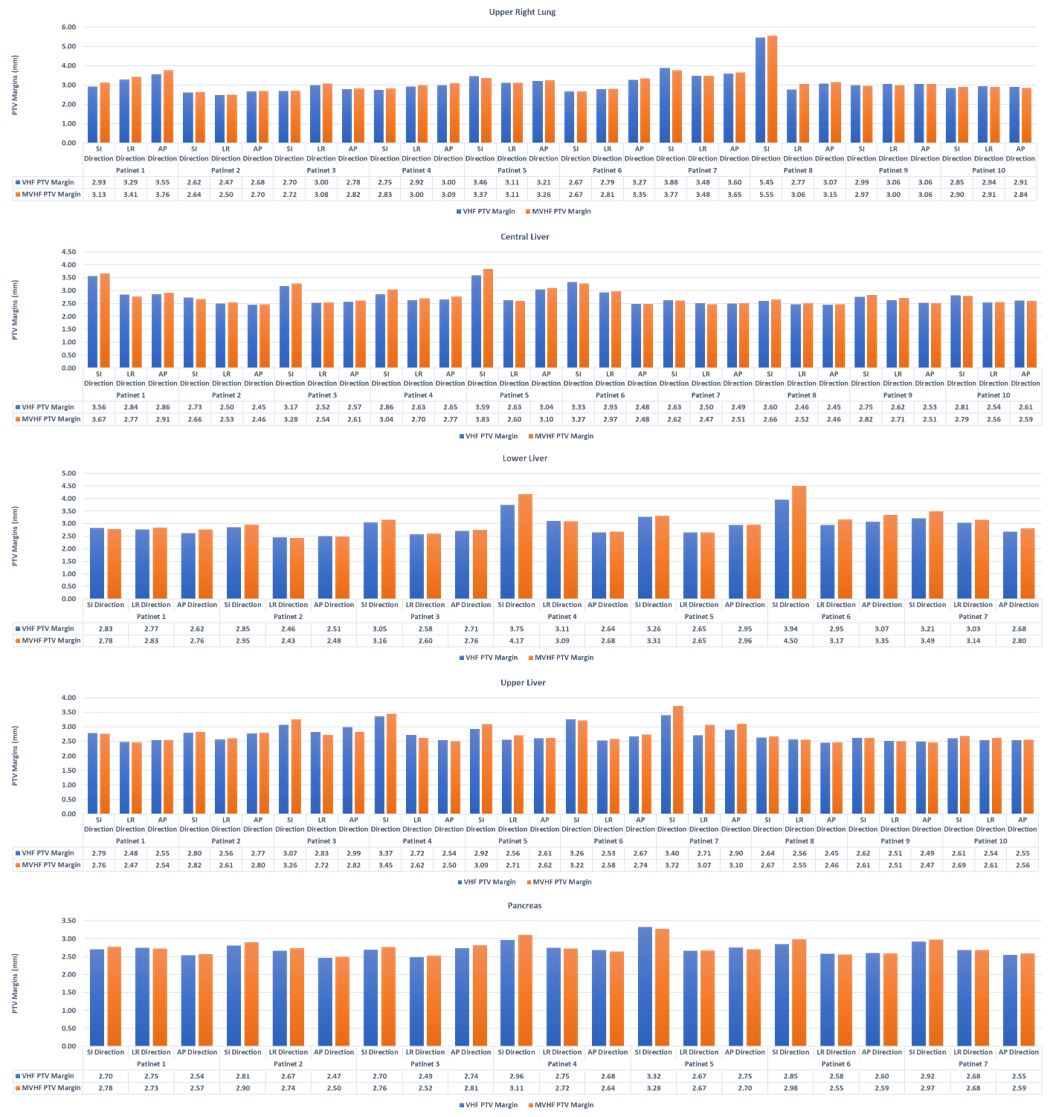

Supplement: Supplementary file 2 — Supporting Information [file ACM2-24-e13975-s003.tif]
