# Supplementary material for: A retrospective multi‐center feasibility study of a new PTV margin estimation approach for moving targets using CyberKnife log files
Source: J Appl Clin Med Phys. 2023 Apr 1;24(7):e13975. doi: 10.1002/acm2.13975 (PMC10338771; doi:10.1002/acm2.13975)
Supplement: Supplementary file 3 — Supporting Information [file ACM2-24-e13975-s002.pdf]

Supplementary Table 1. The average correlation and covariance values between the model and prediction errors for different tumor locations and CyberKnife (CK) versions.

CyberKnife Type			Tumor sites												
			Chest Wall	Lung Apex Left	Lower Left Lung	Upper Left Lung	Lower Right Lung	Central Right Lung	Upper Right Lung	Central Left Lung	Liver	Central Liver	Upper Liver	Lower Liver	Pancreas
G3 Version 6.2.3 *	Correlation (C,P)	SI	0.09±0.09	-0.05±0.14	0.14±0.09	0.05±0.23	0.11±0.18	0.08±0.13	0.05±0.18	-	0.01±0.25	-	-	-	0.02±0.13
		LR	-0.06±0.18	-0.07±0.06	-0.06±0.13	0.02±0.18	-0.05±0.30	-0.02±0.15	-0.007±0.18	-	0.13±0.09	-	-	-	0.004±0.15
		AP	-0.08±0.10	0.03±0.13	-0.03±0.26	0.07±0.16	0.08±0.16	0.06±0.15	-0.03±0.23	-	0.06±0.06	-	-	-	-0.01±014
	Covarinace (C,P)	SI	0.05±0.48	-0.03±0.04	0.28±0.04	0.12±0.19	0.06±0.07	0.07±0.03	0.15±0.07	-	0.12±0.06	-	-	-	0.04±0.05
		LR	-0.04±0.04	-0.01±0.01	-0.17±0.12	0.02±0.09	-0.33±0.04	-0.03±0.03	-0.13±0.20	-	0.19±0.09	-	-	-	0.07±0.08
		AP	-0.01±0.01	0.07±0.04	-0.25±0.13	0.06±0.06	0.12±0.13	0.12±.0.02	-0.12±0.10	-	0.18±0.14	-	-	-	-0.02±0.15
VSI Version 8.5	Correlation (C,P)	SI	-	-	0.01±0.19	-0.15±0.29	-0.02±0.18	-0.001±0.23	-0.01±0.24	-0.03±0.28	-	-0.05±0.28	0.03±0.29	-0.02±0.28	0.00±0.24
		LR	-	-	0.06±0.18	0.11±0.29	-0.04±0.20	-0.02±0.27	0.02±0.21	0.09±0.19	-	-0.01±0.28	0.03±0.22	-0.13±0.25	-0.02±0.26
		AP	-	-	0.001±0.18	-0.03±0.26	-0.08±0.17	0.10±0.26	-0.05±0.28	-0.04±0.25	-	-0.08±0.28	-0.06±0.32	-0.04±0.29	-0.10±0.30
	Covarinace (C,P)	SI	-	-	0.001±0.04	-0.02±0.06	-0.01±0.13	-0.01±0.09	-0.001±0.02	-0.001±0.06	-	-0.01±0.06	0.01±0.08	-0.05±0.21	0.01±0.04
		LR	-	-	0.001±0.02	0.03±0.09	-0.03±0.13	-0.02±0.08	0.01±0.03	0.01±0.04	-	-0.001±0.02	0.00±0.03	-0.02±0.03	-0.001±0.01
		AP	-	-	0.0012±0.04	-0.001±0.03	-0.02±0.10	0.08±0.20	-0.001±0.02	-0.02±0.16	-	-0.01±0.02	-0.001±0.03	-0.001±0.05	-0.01±0.03
VSI Version 9.5	Correlation (C,P)	SI	-	-	0.05±0.17	-0.01±0.22	0.24±0.16	-	-0.01±0.19	-	-	0.06±0.21	0.01±0.18	-	-
		LR	-	-	-0.01±0.25	0.001± 0.25	-0.18±0.25	-	-0.07±0.23	-	-	0.02±0.17	0.01±0.22	-	-
		AP	-	-	0.07±0.26	0.10±0.22	-0.02±0.26	-	0.02±0.22	-	-	-0.11±0.22	0.08±0.22	-	-
	Covarinace (C,P)	SI	-	-	0.03±0.07	-0.001±0.04	0.01±0.01	-	-0.01±0.06	-	-	0.00±0.01	0.00±0.01	-	-
		LR	-	-	-0.05±0.08	0.00±0.01	-0.01±0.01	-	-0.01±0.06	-	-	0.00±0.04	0.04±0.16	-	-
		AP	-	-	0.07±0.14	0.00±0.01	-0.001±0.01	-	0.001±0.02	-	-	-0.01±0.02	0.01±0.03	-	-

All value is based on Mean value ± Standard Deviation.
SI: Superior-Inferior, LR=Left-Right (LR), and AP= Anterior-Posterior.
* Reported by Samadi et al. [15].

Supplementary Table 2. The mean value and standard deviation of model and prediction errors in each direction for different tumor locations and CyberKnife versions.

CyberKnife Type			Tumor sites												
			Chest Wall	Lung Apex Left	Lower Left Lung	Upper Left Lung	Lower Right Lung	Central Right Lung	Upper Right Lung	Central Left Lung	Liver	Central Liver	Upper Liver	Lower Liver	Pancreas
G3 Version 6.2.3 *	Prediction Error [mm]	SI	-0.01±0.59	-0.02±0.98	-0.09±1.81	-0.09±1.12	-0.12±0.69	0.01±0.65	-0.01±1.05	-	0.06±0.49	-	-	-	0.07±0.96
		LR	-0.15±0.50	-0.08±0.34	0.03±1.39	0.00±0.90	0.17±1.23	0.02±0.64	0.09±1.23	-	0.13±1.02	-	-	-	0.00±0.93
		AP	0.07±0.49	0.12±1.00	-0.07±1.42	0.00±0.90	-0.03±0.71	0.03±0.62	0.14±1.16	-	-0.40±1.08	-	-	-	0.01±0.96
	Model Error [mm]	SI	0.23±0.33	0.18±0.53	0.56±0.76	0.40±0.56	0.29±0.43	0.29±0.43	0.37±0.51	-	0.56±0.65	-	-	-	0.32±0.43
		LR	0.16±0.42	0.14±0.35	0.60±0.86	0.35±0.45	0.83±1.14	0.41±0.41	0.49±0.63	-	0.71±0.71	-	-	-	0.51±0.56
		AP	0.16±0.52	0.14±0.40	0.59±0.93	0.41±0.51	0.73±0.79	0.33±0.36	0.41±0.62	-	0.50±0.54	-	-	-	0.49±0.52
VSI Version 8.5	Prediction Error [mm]	SI	-	-	-0.13±1.16	-0.11±0.91	0.03±1.70	-0.04±1.26	0.12±1.02	-0.04±0.93	-	-0.17±1.00	0.002±1.01	-0.06±1.27	0.02±0.98
		LR	-	-	-0.24±1.07	0.02±1.06	-0.27±1.47	-0.14±1.24	-0.10±1.10	-0.10±1.05	-	-0.09±0.54	-0.05±0.58	-0.13±0.68	-0.07±0.60
		AP	-	-	0.05±0.97	0.08±0.82	-0.03±1.33	0.04±1.14	0.09±0.97	0.02±1.00	-	0.03±0.53	0.002±0.49	-0.18±0.68	-0.07±0.64
	Model Error [mm]	SI	-	-	0.26±0.22	0.12±0.11	0.25±0.24	0.17±0.17	0.10±0.08	0.10±0.11	-	0.19±0.12	0.23±0.15	0.41±0.40	0.23±0.13
		LR	-	-	0.11±0.13	0.08±0.11	0.14±0.17	0.15±0.15	0.11±0.10	0.09±0.10	-	0.14±0.09	0.16±0.11	0.18±0.17	0.10±0.06
		AP	-	-	0.23±0.20	0.11±0.10	0.29±0.28	0.25±0.24	0.10±0.09	0.13±0.14	-	0.21±0.14	0.25±0.19	0.30±0.31	0.18±0.11
VSI Version 9.5	Prediction Error [mm]	SI	-	-	-0.41±1.38	-0.07±0.96	-0.04±0.80	-	-0.13±0.94	-	-	-0.04±0.53	-0.03±0.58	-	-
		LR	-	-	-0.10±1.03	-0.06±0.64	-0.16±0.93	-	0.15±0.81	-	-	-0.06±1.16	-0.05±1.10	-	-
		AP	-	-	-0.09±1.14	0.10±0.79	0.03±0.53	-	-0.03±0.69	-	-	-0.07±0.58	-0.04±0.62	-	-
	Model Error [mm]	SI	-	-	0.15±0.20	0.05±0.07	0.03±0.07	-	0.12±0.14	-	-	0.09±0.10	0.13±0.12	-	-
		LR	-	-	0.21±0.46	0.05±0.05	0.14±0.11	-	0.16±0.14	-	-	0.24±0.18	0.24±0.23	-	-
		AP	-	-	0.24±0.45	0.03±0.06	0.15±0.12	-	0.14±0.11	-	-	0.26±0.19	0.22±0.20	-	-

All value is based on Mean value ± Standard Deviation.
SI: Superior-Inferior, LR=Left-Right (LR), and AP= Anterior-Posterior.
* Reported by Samadi et al. [15].

Supplementary Table 3. The uncertainty of model and prediction errors in each direction for different tumor locations and CyberKnife versions.

CyberKnife Type			Tumor sites												
			Chest Wall	Lung Apex Left	Lower Left Lung	Upper Left Lung	Lower Right Lung	Central Right Lung	Upper Right Lung	Central Left Lung	Liver	Central Liver	Upper Liver	Lower Liver	Pancreas
G3 Version 6.2.3 *	Model Uncertainty [mm]	SI	1.24	1.99	3.86	2.43	1.53	1.38	2.29	-	1.05	-	-	-	2.06
		LR	1.15	0.77	3.04	1.97	2.69	1.39	2.64	-	2.28	-	-	-	2.07
		AP	1.10	2.12	3.09	1.96	1.55	1.33	2.53	-	2.56	-	-	-	2.05
	Prediction Uncertainty [mm]	SI	0.90	1.24	2.09	1.52	1.14	1.14	1.40	-	1.87	-	-	-	1.30
		LR	1.01	0.84	2.32	1.26	3.12	1.24	1.75	-	2.12	-	-	-	1.63
		AP	1.20	0.94	2.45	1.43	2.31	1.04	1.64	-	1.58	-	-	-	1.52
VSI Version 8.5	Model Uncertainty [mm]	SI	-	-	2.64	2.15	3.70	2.90	2.39	2.07	-	2.32	2.32	2.88	2.21
		LR	-	-	2.43	2.40	3.31	2.84	2.49	2.31	-	1.21	1.29	1.58	1.30
		AP	-	-	2.22	1.87	2.86	2.62	2.23	2.25	-	1.16	1.10	1.56	1.47
	Prediction Uncertainty [mm]	SI	-	-	0.69	0.33	0.74	0.50	0.25	0.31	-	0.43	0.53	1.20	0.49
		LR	-	-	0.38	0.29	0.48	0.44	0.31	0.30	-	0.32	0.39	0.51	0.22
		AP	-	-	0.63	0.30	0.84	0.74	0.27	0.42	-	0.48	0.63	0.93	0.40
VSI Version 9.5	Model Uncertainty [mm]	SI	-	-	3.17	2.05	1.70	-	2.09	-	-	1.15	1.25	-	-
		LR	-	-	2.18	1.41	2.07	-	1.85	-	-	2.52	2.38	-	-
		AP	-	-	2.60	1.69	1.14	-	1.53	-	-	1.26	1.35	-	-
	Prediction Uncertainty [mm]	SI	-	-	0.56	0.18	0.18	-	0.39	-	-	0.30	0.37	-	-
		LR	-	-	1.14	0.14	0.36	-	0.43	-	-	0.60	0.70	-	-
		AP	-	-	1.14	0.15	0.38	-	0.37	-	-	0.63	0.62	-	-

SI: Superior-Inferior, LR=Left-Right (LR), and AP= Anterior-Posterior.
* Reported by Samadi et al. [15].

Supplementary Table 4. Pearson and distance correlation for different CyberKnife versions, tumor locations, and directions.

Tumor Sites		CK G3 version 6.2.3		CK VSI Version 8.5		CK VSI Version 9.5	
		Pearson Correlation	Distance Correlation	Pearson Correlation	Distance Correlation	Pearson Correlation	Distance Correlation
Lower Left Lung	SI	0.14±0.09	0.26±0.06	0.01±0.19	0.26±0.09	0.05±0.17	0.27±0.13
	LR	-0.06±0.13	0.24±0.08	0.06±0.18	0.25±0.08	-0.01±0.25	0.31±0.11
	AP	-0.03±0.26	0.33±0.12	0.001±0.18	0.26±0.10	0.07±0.26	0.26±0.08
Upper Left Lung	SI	0.05±0.23	0.28±0.1	-0.15±0.29	0.35±0.14	-0.01±0.22	0.28±0.10
	LR	0.02±0.18	0.27±0.09	0.11±0.29	0.34±0.08	0.001±0.25	0.26±0.15
	AP	0.07±0.16	0.25±0.09	-0.03±0.26	0.36±0.13	0.10±0.22	0.24±0.12
Lower Right Lung	SI	0.11±0.18	0.25±0.11	-0.02±0.18	0.27±0.09	0.24±0.16	0.26±0.19
	LR	-0.05±0.03	0.34±0.15	-0.04±0.20	0.27±0.09	-0.18±0.25	0.28±0.09
	AP	0.08±0.16	0.25±0.08	-0.08±0.17	0.26±0.11	-0.02±0.26	0.24±0.15
Upper Right Lung	SI	0.05±0.18	0.25±0.11	-0.01±0.24	0.32±0.11	-0.01±0.19	0.28±0.13
	LR	-0.007±0.18	0.30±0.14	0.02±0.21	0.34±0.14	-0.07±0.23	0.26±0.13
	AP	-0.03±0.23	0.26±0.12	-0.05±0.28	0.30±0.12	0.02±0.22	0.25±0.12

SI: superoinferior, LR=left-right (LR), and AP=anteroposterior directions.

Supplementary Table 5.1. The maximum and minimum correlation values between the model and prediction errors, maximum and minimum PTV Margin based on MVHF, and relative error between the Van Herk and the Modified Van Herk PTV Margin in the CyberKnife G3-version 6.2.3.

Tumor sites	Correlation (Mod, Pred)						Maximum and Minimum PTV Margin based on MVHF [mm]						Relative error between PTV Margin based on VHF and MVHF with maximum and minimum correlation (%)					
	SI		LR		AP		SI		LR		AP		SI		LR		AP	
	Min	Max	Min	Max	Min	Max	Min	Max	Min	Max	Min	Max	Min	Max	Min	Max	Min	Max
Chest Wall	0.0016	0.26	-0.36	0.125	-0.23	0.05	2.65	2.77	2.54	2.80	2.70	2.97	0.03	4.42	-6.86	2.38	-7.56	1.55
Lung Apex Left	-0.19	0.14	-0.01	-0.14	-0.11	0.19	3.93	4.29	3.51	3.56	3.95	4.23	-4.96	3.65	-0.09	-1.46	-2.55	4.41
Lower Left Lung	-0.017	0.29	-0.40	0.09	-0.42	0.31	4.84	5.42	4.19	4.70	4.12	5.21	-0.66	11.25	-9.02	2.03	-13.17	9.72
Upper Left Lung	-0.44	0.55	-0.31	0.36	-0.18	0.48	3.75	4.99	3.74	4.59	3.90	4.75	-12.79	15.99	-9.53	11.07	-5.62	14.99
Lower Right Lung	-0.099	0.49	-0.45	0.62	-0.24	0.35	2.87	3.34	2.85	4.80	2.77	3.36	-2.65	13.12	-22.44	30.92	-7.89	11.51
Central Right Lung	-0.083	0.38	-0.41	0.18	-0.22	0.36	2.91	3.23	2.52	2.90	2.58	2.90	-1.93	8.83	-9.44	4.14	-4.56	7.47
Upper Right Lung	-0.2	0.49	-0.45	0.32	-0.49	0.42	3.07	4.21	3.03	4.64	2.86	3.81	-9.76	23.92	-23.80	16.93	-15.27	13.09
Liver	-0.39	0.35	-0.02	0.24	-0.01	0.17	2.63	3.11	3.25	3.43	3.21	3.42	-8.80	7.90	-0.42	5.08	-0.41	6.28
Pancreas	-0.23	0.28	-0.28	0.32	-0.32	0.28	3.86	4.26	3.97	4.24	3.84	4.44	-4.44	5.41	-3.07	3.51	-7.69	6.73

SI: superoinferior, LR=left-right (LR), and AP=anteroposterior directions.
C = Correlation Error
P = Prediction Error

Supplementary Table 5.2. The maximum and minimum correlation values between the model and prediction errors, maximum and minimum PTV Margin based on MVHF, and relative error between the Van Herk and the Modified Van Herk PTV Margin in the CyberKnife VSI-version 8.5.

Tumor sites	Correlation (Mod, Pred)						Maximum and Minimum PTV Margin based on MVHF [mm]						Relative error between PTV Margin based on VHF and MVHF with maximum and minimum correlation (%)					
	SI		LR		AP		SI		LR		AP		SI		LR		AP	
	Min	Max	Min	Max	Min	Max	Min	Max	Min	Max	Min	Max	Min	Max	Min	Max	Min	Max
Central Left Lung	-0.25	0.25	-0.33	0.24	-0.16	0.18	4.00	5.19	3.68	5.83	4.24	4.64	-1.64	2.87	-3.69	4.15	-1.43	1.10
Lower Left Lung	-0.32	0.37	-0.43	0.36	-0.29	0.34	4.02	4.35	3.90	5.90	3.65	4.62	-7.98	6.53	-4.63	3.82	-1.53	1.61
Upper Left Lung	-0.29	0.39	-0.53	0.29	-0.58	0.54	3.75	4.91	3.88	4.24	4.05	5.25	-4.04	3.39	-2.83	1.74	-2.63	2.17
Lower Right Lung	-0.34	0.22	-0.52	0.13	-0.32	0.27	3.59	3.78	3.62	3.87	4.73	4.90	-5.99	4.40	-3.53	2.94	-3.06	0.61
Central Right Lung	-0.28	0.53	-0.49	0.25	-0.28	0.53	4.08	4.27	2.67	4.31	3.04	3.24	-2.62	2.37	-0.89	7.49	-5.71	0.64
Upper Right Lung	-0.45	0.30	-0.54	0.59	-0.36	0.39	2.90	5.48	2.81	3.08	3.25	3.61	-4.93	3.53	-1.02	1.59	-0.87	1.71
Central Liver	-0.72	0.66	-0.55	0.64	-0.65	0.36	3.38	3.48	2.48	2.94	2.69	2.97	-3.23	1.68	-2.00	1.51	-2.22	1.69
Lower Liver	-0.21	0.70	-0.16	0.25	-0.53	0.36	2.90	3.29	2.86	3.00	2.52	2.59	-12.22	2.49	-3.66	3.32	-3.49	0.39
Upper Liver	-0.25	0.37	-0.74	0.28	-0.27	0.40	3.29	3.58	2.55	2.81	2.82	3.05	-3.14	5.41	-1.41	3.47	-2.86	2.12
Pancreas	-0.42	0.57	-0.52	0.45	-0.46	0.35	2.75	3.22	2.58	2.70	2.73	2.77	-2.88	1.89	-3.38	0.75	-0.98	0.69

SI: superoinferior, LR=left-right (LR), and AP=anteroposterior directions.
C = Correlation Error
P = Prediction Error

Supplementary Table 5.3. The maximum and minimum correlation values between the model and prediction errors, maximum and minimum PTV Margin based on MVHF, and relative error between the Van Herk and the Modified Van Herk PTV Margin in the CyberKnife VSI-version 9.5.

Tumor sites	Correlation (Mod, Pred)						Maximum and Minimum PTV Margin based on MVHF [mm]						Relative error between PTV Margin based on VHF and MVHF with maximum and minimum correlation (%)					
	SI		LR		AP		SI		LR		AP		SI		LR		AP	
	Min	Max	Min	Max	Min	Max	Min	Max	Min	Max	Min	Max	Min	Max	Min	Max	Min	Max
Lower Left Lung	-0.44	0.41	-0.26	0.50	-0.23	0.19	3.74	4.66	4.89	5.06	3.99	4.72	-5.34	10.10	-2.82	2.72	-0.65	1.85
Upper Left Lung	-0.24	0.55	-0.36	0.52	-0.44	0.34	3.58	4.13	3.58	3.63	4.59	4.96	-2.45	3.29	-2.05	0.88	-1.07	1.41
Lower Right Lung	-0.55	0.12	-0.36	0.32	-0.14	0.15	2.61	2.69	2.51	2.79	3.09	3.10	-5.50	3.07	-2.91	2.38	-2.15	0.51
Upper Right Lung	-0.63	0.40	-0.42	0.35	-0.15	0.38	3.15	4.30	2.63	2.83	3.11	3.81	-4.83	2.84	-1.66	1.86	-1.32	1.49
Central Liver	-0.38	0.36	-0.39	0.43	-0.32	0.19	2.79	3.39	2.69	2.76	2.71	2.79	-1.87	1.74	-2.51	2.45	-1.40	1.15
Upper Liver	-0.44	0.26	-0.53	0.46	-0.35	0.21	2.79	3.91	2.71	2.64	2.79	2.83	-2.85	8.30	-2.02	2.87	-1.71	1.83

SI: superoinferior, LR=left-right (LR), and AP=anteroposterior directions.
C = Correlation Error
P = Prediction Error
